# Exploring Less Invasive Visual Surveys to Assess the Spatial Distribution of Endangered Mediterranean Trout Population in a Small Intermittent Stream

**DOI:** 10.3390/biology12071000

**Published:** 2023-07-13

**Authors:** Francesco Palmas, Paolo Casula, Francesco Curreli, Cinzia Podda, Serenella Cabiddu, Andrea Sabatini

**Affiliations:** 1 Department of Life and Environmental Sciences, University of Cagliari, Via Fiorelli 1, 09126 Cagliari, Italy; c.podda1@gmail.com (C.P.); cabiddus@unica.it (S.C.); asabati@unica.it (A.S.); 2 Agenzia Forestas, Servizio Tecnico, Viale Merello 86, 09124 Cagliari, Italy; pcasula@forestas.it (P.C.); f.curreli@hotmail.it (F.C.)

**Keywords:** intermittent rivers, Mediterranean trout, monitoring, visual methods, non-invasive methods, trout occupancy

## Abstract

**Simple Summary:**

This study explores the use of alternative and non-harmful methods for monitoring endangered Mediterranean trout in small intermittent streams. Specifically, two visual survey techniques were compared: a visual survey from streambanks and an underwater visual survey using underwater cameras. The main objective was to assess the effectiveness of detecting patterns in fish occupancy in relation to a set of environmental factors. The comparison revealed that underwater camera surveys performed similarity to visual surveys from streambanks during low-flow regimes. However, visual surveys from streambanks were less effective during the highest flow regimes. The volume of pools and the percentage cover of submerged macrophytes were found to be significant environmental variables affecting fish detection probability using underwater cameras. On the other hand, the analysis of data from visual observations from streambanks indicated a clear impact of high turbulence rates on pool occupancy. In conclusion, these findings demonstrate the utility of visual methods in describing the occupancy patterns of Mediterranean trout in small streams.

**Abstract:**

Monitoring the conservation status of endangered freshwater fish using less invasive methods poses challenges for ecologists and conservationists. Visual surveys have been proposed as an alternative to electrofishing, which is a standard methodology that can cause injuries, physiological stress and post-release mortality in organisms. To test the efficacy of visual methods, a study was conducted in an intermittent stream of Sardinia (Italy). Two visual methods were employed: a visual survey from streambanks (VSS) and an underwater visual survey (UVS) using cameras. The aims of this study were (1) to compare the effectiveness of these methods in detecting patch occupancy patterns and (2) to investigate the effect of environmental variables on the detection probability of Mediterranean native trout. Environmental variables characterizing pool habitats were recorded, and generalized linear models (GLMs) were employed to assess the correlation between these variables and trout presence/absence. GLM analysis revealed that UVS had higher detection probability with larger pool volume, whereas submerged macrophytes negatively affected detection probability. Detection from streambanks (VVS) was negatively affected by a high turbulence rate. In conclusion, our study suggests the utility of visual methods to describe patterns of patch occupancy of Mediterranean trout. However, methods can be differently affected by environmental variables. Therefore, monitoring programs using these methods should consider these factors to ensure a reliable description of within-stream trout distribution in intermittent streams.

## 1. Introduction

Monitoring the status of imperiled freshwater fish using effective and non-invasive methods is a crucial concern for ecologists. Fish populations that are worthy of conservation require monitoring over time and space to identify changes in occupancy and abundance or the documentation of long-term temporal changes in entire communities [1,2,3,4]. Sampling methods for fish monitoring are numerous and usually involve the application of capture and non-capture techniques that vary on the basis of the habitat, target species and aims of the study [5].

In small streams, one of the most common capture techniques is multiple-pass removal electrofishing, because it is generally assumed that this method provides reliable estimates of the relative abundance of fish [6,7,8]. Although electrofishing may provide detailed information that is not obtainable via non-capture techniques [5], such sampling methods can cause injures, physiological stress and sometimes post-release mortality for both target and non-target organisms [9,10,11,12]. Detrimental effects of electrofishing should be avoided in the monitoring protocol developed within protected areas and for rare or endangered species [13,14]. Furthermore, headwater refuges often are in remote locations with little to no road access, where bulky conventional sampling equipment, such as backpack electrofisher, may be impossible to transport [15,16].

For these reasons, non-harmful and low-cost alternative methods for monitoring endangered fish populations have been explored. These include environmental DNA (eDNA) techniques [17,18,19] and visual observation techniques [15,20,21,22]. These methods have gained recent attention and are being tested as viable alternatives for monitoring endangered fish populations [23].

Visual estimation methods in streams have been undertaken by snorkeling [21,24,25,26], using underwater cameras [27,28,29,30] and from streamside visual surveys [15,31,32,33]. The successful use of visual observation to detect the presence of fish species has been shown, even if detection probabilities may be affected by habitat conditions [16,26,31,34,35] and fish behavior related to different life stages [21,36].

In Mediterranean streams, the native Mediterranean trout stands as one of the most imperiled fish species, facing drastic population declines over the past few decades [37]. Nevertheless, the taxonomic classification of the native Mediterranean trout found in Sardinia remains a topic of ongoing research and debate [38,39,40,41]. It is currently suggested that the trout population in Sardinia may represent an undescribed species, warranting a distinct scientific name [42]. At present, the species is listed as critically endangered, with the name of *Salmo ghigii*, in the national Red List of the International Union for Conservation of Nature (IUCN) [43], and the same population is listed (as *Salmo macrostigma* (Duméril, 1858)) in Annex II of the Habitats Directive (92/43/EEC). The main threats to the species are represented by habitat fragmentation, water pollution, climate change [44,45] and the introduction of domestic trout [46,47,48,49]. Additionally, in Mediterranean rivers, the concurrent and interacting effects of climate-driven changes result in variations in flow rates and thermal regimes that enhance stream fragmentation [50,51,52,53]. Though isolation through natural and/or artificial barriers may represent a key factor for securing the persistence of native trout populations [46,54,55,56,57], fragmentation makes them vulnerable to environmental disturbances [58] and inbreeding depression [59,60,61]. Indeed, native Sardinian brown trout appear to be subdivided into very small, isolated and highly genetically differentiated populations living in peculiar and extreme environments [54,55].

Fragmentation and inbreeding appear even more crucial in the southern Mediterranean region, where most rivers have intermittent water flow regimes [62]. To preserve the native gene pools remaining in Sardinia Island basins, the autonomous region designated several river segments as ‘genetic sanctuaries’ (sensu [63]), where fishing and stocking activities are totally banned (DR n.314/Dec.A9 07.02.2019). Key for conservation planning is determining where remaining small and fragmented populations will persist, and, in this context, the long-term monitoring of Mediterranean trout populations and occurrence patterns requires non-harmful methods.

Here, we consider that long-term monitoring of small and endangered fish populations should be based on non-invasive methods, which can be affected by seasonally varying environmental conditions. Therefore, we investigate how monitoring based on underwater cameras and streamside surveys can be used to assess the distribution of Mediterranean native trout. Specifically, (1) we compare the relative effectiveness of two methods in detecting patterns of patch occupancy of Mediterranean native trout during low- and high-flow regimes, and (2) we investigate the effect of a set of environmental variables that could affect the detection probability of fish for each method.

## 2. Materials and Methods

### 2.1. Study Area

The Sardinian climate, like other Mediterranean areas, follows a clear pattern with two distinct seasons: severe drought in summer and a rainy autumn/winter, resulting in irregular flow and significant seasonal hydrological fluctuations, exacerbated by the presence of numerous dams [64,65]. Consequently, the hydrographic network in Sardinia is characterized by a reduced number of perennial rivers and a prevalence of intermittent streams [66].

**Figure 1:**
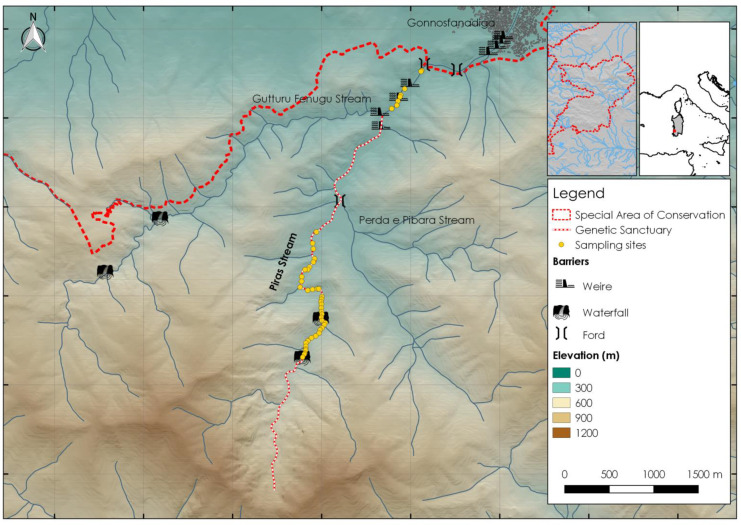
Study area and sampling stations of the investigated pools.

The study was conducted in the Piras River, a headwater stream located in the south-west of Sardinia (Italy) (Figure 1).

The stream flows north for 6 km through the Natura 2000 Special Area of Conservation of Monte Linas-Marganai (ITB041111, Habitats Directive 92/43/EEC) and hosts only two native fish species: the stickleback (*Gasterosteus aculeatus*) and the native Mediterranean trout (*Salmo ghigii* as reported in the national Red List of International Union for Conservation of Nature (IUCN) [43]). The headwater section of the stream, located approximately 4.3 km from the stream source, has been designated as a genetic sanctuary (DR n.314/Dec.A9 07.02.2019). The stream has an intermittent regime, with water availability dependent on precipitation events, and fragmented pools consistently observed during periods of low flow. During the summer, surface flow is virtually absent, and the stream features long stretches of dry areas with small, isolated pools. Furthermore, the lower section of the stream is characterized by various barriers to river continuity, including human-made weirs and fords. Riparian vegetation is mainly composed of holm oak (*Quercus ilex* L., 1753), alder (*Alnus glutinosa* Gaertn., 1790), royal fern (*Osmunda regalis* L., 1753), elmleaf blackberry (*Rubus ulmifolius* Schott, 1818) and oleander (*Nerium oleander* L., 1753).

### 2.2. Data Collection

Field samplings were conducted in 47 pools distributed along a 7 km stretch of the stream, and they were replicated during two sampling seasons corresponding to the highest flow regime (HFR) and the lowest flow regime (LFR). The pool was defined as a habitat unit with a minimum depth of 20 cm and characterized by a slow current. In the case of adjacent habitat units, we selected pools with a minimum distance of 50 m between each other. Since our purpose was to investigate the distribution of trout in stable habitats, we deliberately avoided sampling stream stretches predominately characterized by turbulent riffle during the HFR and dry habitat (lacking surface water) during LFR.

The visual survey from streambanks (VSS) was conducted by two experienced observers who walked along the stream on opposite banks simultaneously. At each pool, the observers stopped, remained stationary and counted the trout. To avoid double counts, the observers communicated with each other and shared the positions of all observed fish. After a duration of 3 min, the total counts were recorded. Following the visual observation, an underwater camera was promptly placed in the pool, and the set of habitat variables listed in Table S1 were also measured. The underwater visual survey (UVS) was conducted using action cameras (Apeman Action Cam A100, Longgang, Shenzhen, China) mounted on a stable stand (Sabrent Magnetic Support, Los Angeles, CA, USA). The cameras were positioned facing upstream in the pool to maximize the field of view. The observers then moved away from the streambanks, proceeded upstream to the next pool, and repeated this observation process until the entire sampling area was covered. The underwater camera continuously recorded video footage for a minimum of 30 min. The videos were captured at a resolution of 1080p, with a frame rate of 30 frames per second. The camera had an ultra-wide field of view and a screen resolution of 1920 × 1080. A recording time of 30 min is considered sufficient for the reliable monitoring of fish populations in small streams [22,27].

### 2.3. Environmental Variables

Data were recorded for temporal and habitat variables that were hypothesized to influence the occupancy of Mediterranean trout and/or detection probability for each visual method employed (Table S2), as follows. The temporal variable describes the sampling season and corresponds with the highest and lowest flow regime (HFR and LFR, respectively). Variables describing the size of pools were measured using a rolmeter and include the pool length (m), the average pool width (m), the maximum value of the pool depth (m) and the pool volume (m^3^). Specifically, the length of the pool was measured, and subsequently, the pool was divided into three transects of equal length. The width (m) of each transect was measured in order to obtain the pool width. Covariates describing pool size were included, because they may affect habitat selection by brown trout [67], as well as species detection probability.

At each pool, water temperature (T_H_2_O, °C) and dissolved oxygen concentration (O_2__mgl, mg/L) were measured using a multiparameter probe (InSitu smarTROLL Multiparameter Handheld, Fort Collins, CO, USA). These measurements were included because the presence of brown trout and microhabitat selection can be influenced by water temperature and oxygen concentration [45,67,68]. In addition, the water turbulence rate was visually estimated and categorized as low (LTR), medium (MTR) or high (HTR). Turbulence was defined as abrupt changes in water velocity typically observed at points of gradient change (small cascades), near physical obstructions to flow (wood or boulders) and along irregular banks. Turbulence was considered as LTR when there was no current or wind speed causing a disrupted water surface, HTR when the current or wind speed were high enough to create choppy water or riffles and MTR when the water velocity was moderate and did not pose significant difficulties to observing the stream underwater. Water turbidity (Turbidity) was recorded using a turbidity meter (NTU) (AQUALYTIC^®^ AL255T-IR, Pretoria, South Africa). Turbulence rate and turbidity (NTU) were measured because elevated levels of these parameters can reduce visibility and potentially have a negative impact on the detection probability of fish [15,16,31].

The proportion (%) of substrate and organic cover in the entire pool streambed were also visually estimated. The substrate was classified according to modified categories from Platts et al., 1883 [69], including bedrock, boulder (more than 304.0 mm), cobble (76.1–304.0 mm), gravel (4.8–76.0 mm), sand (0.8–4.7 mm) and silt (particle size < 0.8 mm). Organic cover was characterized using a modified classification of Hering et al., 2004 [70], as emergent macrophytes, submerged macrophytes, periphyton, green algae, roots, riparian vegetation, XYLAL (dead wood) and FPOM (fine particulate organic matter). Both substrate features and organic cover can be influential factors in brown trout habitat selection [67,71]. Among the organic and substrate features, submerged macrophytes, green algae, roots and riparian vegetation, as well as the percentage cover of boulders, have been taken into account. This is because it has been hypothesized that the complexity of organic and substrate features could potentially have a negative impact on the effectiveness of visual methods [15,34].

### 2.4. Data Analyses

Detection rates were calculated as the percentage of pools in which the presence of trout was recorded using each visual method and sampling season. Differences in detection rates between the sampling methods were analyzed with G-tests of independence (*p* < 0.05). Distribution maps of trout presence and absence data for each visual method and sampling season were generated using Geographic Information System (GIS) software (Quantum GIS Desktop, version 2.18.3).

For general descriptive analyses, each sampling season (HFR and LFR) was analyzed separately. For continuous habitat variables, the range, mean and standard error (SE) were estimated. Differences in habitat variables between the sampling seasons were tested for normality using the Shapiro test and for homogeneity of variance using the Levene test. As the data did not meet the assumption of normality, a non-parametric Kruskal–Wallis (K-W) test was performed (*p* < 0.05).

For each visual method, the relationships between habitat variables and occurrence of the species were modelled using a logistic regression with generalized linear models (GLM). The GLM models were applied with a binomial error distribution and logit link function. Trout occurrence was coded as a binary response variable (0 = absence, 1 = presence). Only presence/absence data were considered, as the aim was to identify the environmental conditions that might represent a threshold beyond which trout may be undetectable with the visual methods employed.

To assess collinearity among environmental variables, a pairwise scatter plot was generated for comparison of continuous variables. Combinations in which any relevant Sperman’s Rho (ρ > 0.6) was observed in pairs were discarded for the modelling. Following [72], the variance inflation factor (VIF) was also used to check collinearities among the predictive variables, and variables with VIF > 3 were discarded from the analysis. Pairwise scatter plots only capture two-way relationships, whereas the VIF detects high-dimensional collinearity.

Considering the relatively small data set (N = 47), for each sampling method, univariate GLM analysis was first performed, and the effect of a single variable was evaluated at a time. Each univariate model was compared with a null model (M0) that assumed trout occupancy to be constant across flow regimes and pool habitats’ characteristics. Univariate models were compared with M0 by means of Akaike Information Criteria corrected for small samples (AICc) [73] and a likelihood ratio test (LRT). Models that had ΔAICc higher than 2 or that differed significantly from M0 according to the LRT were considered for multivariate analysis.

To further assess the effects supported by the univariate analysis, we ran multivariate automated model selection using the glmulti package [74]. Due to the small data set, interactions among variables were not allowed (level = 1). The relative importance of each predictor was estimated as the sum of Akaike weights (wi) across all the models including it, and we used a cut-off of 0.8 to distinguish the important and unimportant predictors [74]. For the best model, goodness of fit based on deviance was also assessed ((deviance(M0)-deviance(MEffect))/deviance(M0) [75].

## 3. Results

**Figure 2:**
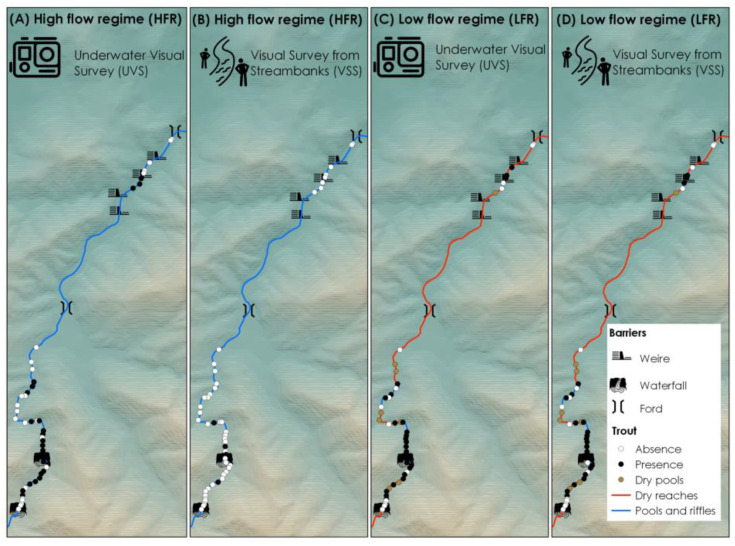
Within-stream distribution of Mediterranean trout in the Piras stream obtained with different visual survey methods (underwater visual survey, UVS and visual survey from streambanks, VSS) and flow regimes (HFR and LFR).

Out of the 47 pools investigated during the HFR, eight were found to be dry during LFR (Figure 2). Pool size and chemical/physical characteristics of water varied considerably between flow regimes (K-W, *p* < 0.05), with the exception of pool depth (Depth) (Table S1). Substrate cover did not show differences between the two regimes, whereas organic cover changed significantly between the two flow regimes, with an increase in XYLAL and FPOM during LFR and a higher percentage of periphyton cover during the HFR (Table S1).

During LFR, the detection rate of trout was quite similar among sampling methods (53% and 51% for UVS and VSS, respectively), whereas during HFR, the observation from streambanks (VSS) revealed a very low value (8%), and underwater cameras showed a value very similar to that of LFR (UVS, 51%). During HFR, the G-test revealed significant differences in the detection rate between UVS and VSS (G = 22.007, df = 1, *p* < 0.001), whereas during LFR, no differences were detected (G = 1.81 × 10^5^, df = 1, *p* = 0.99).

Analysis of multi-collinearity among predictive variables describing pool size revealed strong correlations between pool length (Pool_Length) (ρ = −0.7), average pool width (Pool_Width) (ρ = −0.8), maximum depth (Depth) (ρ = −0.8) and volume of the pool (Volume). We choose to keep the volume of the pool for GLM analysis as the most representative factor that may influence occurrence and detection probability of both visual methods. Since the variables that describe the characteristics of water were strongly correlated (ρ > 0.7), we removed the temperature of water (T_H_2_O) and the concentration of oxygen (O_2__mgl). We kept turbidity (Turbidity), as it could affect fish visibility within pools. Given the analysis of VIF criteria (Table S3), we also removed the temporal variable (Flow_regime) because of strong collinearity with the turbulence rate. Therefore, the variables listed in Table S2 were kept for univariate GLM analysis.

For UVS data, univariate model selection retained three variables: volume of the pools (Volume), the percentage cover of submerged macrophytes (Submerged_macrophyte) and the percentage cover of boulders (Boulder) (Table 1). The first three models were significantly different from M0 (LRT, *p* < 0.01) and also had AICc values lower than M0. The coefficient of the first significant variable was positive, suggesting a greater likelihood of trout detection with UVS when the volume (m^3^) of the pool is higher. The coefficients of the other two variables were negative, indicating that there is a lower likelihood of trout detection when the percentage cover of submerged macrophytes (Submerged_macrophyte) and of boulders (Boulder) are higher. Models ranked from sixth to ninth do not provide any support for hypothesized effects.

**Figure 3:**
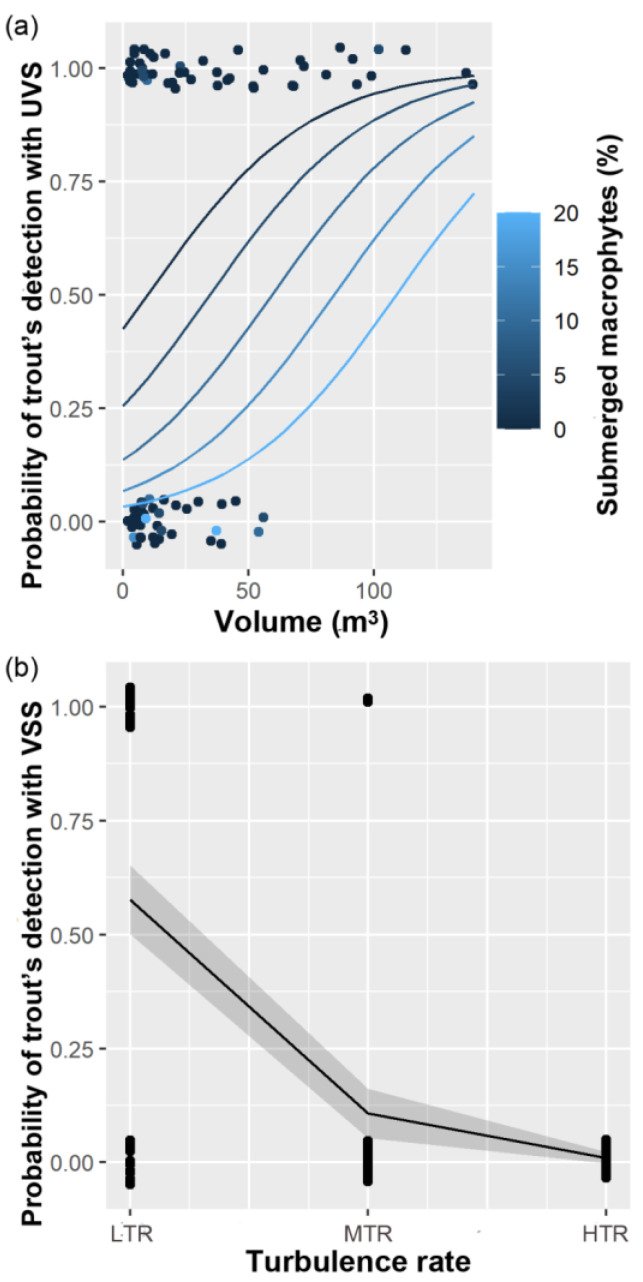
(**a**) Underwater visual surveys (UVS). Estimated probability (solid lines) of trout detection by the volume (m^3^) of pools and percentage cover of submerged macrophytes. The points are the raw binary data of trout detection (N = 86). Best model parameters showed a positive relationship between probability of trout detection with volume of pool (m^3^) ($\mathrm{UVS}=1/\left(1+e^{\left(-\left(0.15*\mathrm{Volume}+1.25\right)\right)}\right)$ and a negative effect of percentage cover of submerged macrophytes ($\mathrm{UVS}=1/\left(1+e^{\left(-\left(0.03*\mathrm{Submerged}\_\mathrm{Macrophites}-1.84\right)\right)}\right)$. (**b**) Visual survey from streambanks (VSS). Estimated probability (solid line) that a trout can be detected with visual observation from streambanks over three levels of water turbulence ($\mathrm{VSS}=1/\left(1+e^{\left(-\left(2.43*\mathrm{Turbulence}\_\mathrm{rate}+2.73\right)\right)}\right)$. Grey shade is 95% confidence band, and dot black points are the raw binary data of trout detection (N = 86).

For UVS data, multi-model selection produced eight models considering different combinations of selected explanatory variables (Table 2). The first two models (ranked first and second) showed ΔAICc < 2 and were not significantly different from each other (LRT, *p* = 0.24). In terms of the relative importance of variables, only pool volume (Volume) and the percentage cover of submerged macrophytes (Submerged_macrophyte) were important predictors (Table 3). The percentage cover of boulders had no support with the multivariate analysis. The most parsimonious model, specifying the effect of Volume and Submerged_macrophyte (ranked first), was selected for inference (deviance explained = 15%). (Table 2). Multivariate model selection thus showed that the probability of trout detection increased in pools with greater volume and decreased with a higher cover of submerged macrophytes (Figure 3a).

For VSS, the univariate model selection supported three variables, Turbulence_rate, turbidity (NTU) and the percentage cover of boulders (Boulders) (Table 1). The first three models (ranked first to third) had AICc values lower than the null model (M0) and were also significantly different on the basis of LRT values (*p* < 0.05). Slope parameters were negative for the turbulence rate and the percentage cover of boulders and positive for turbidity.

Multivariate automated model selection showed similar AICc values for the first three models (ranked first to third) (deviance explained = 26%) (Table 2). The three models are nested and not differentiated on the basis of the LRT test (*p* > 0.05). The turbulence rate showed an importance value > 0.8, the percentage cover of boulders supported only a moderate effect, and the turbidity of water had a weak effect (Table 3). For these reasons, the most parsimonious model, specifying the effect of the turbulence rate (ranked second), was selected (Table 2). The negative slope implies that as the turbulence rate increases, the detection of trout with VSS decreases (Figure 3b).

## 4. Discussion

Our study, although limited to a local scale, provides evidence on the usefulness of visual methods in describing patterns of occurrence in the endangered Mediterranean trout population in small intermittent streams. Indeed, such methods allow for an extensive and detailed investigation of streams in which populations persist. Distributional data gathered during surveys highlight a restricted availability of suitable habitats (approximately 1.5 km) and a patchy distribution across the stream, as observed in other intermittent river systems [76,77]. Specifically, in our study case, most pools in the upstream stretch have trout, whereas in the intermediate stretch, there are few ephemeral and fishless pools, and only a few pools host trout in the downstream stretch.

Moreover, we found that the detection rate of the two visual methods did not change during the low-flow regime (LFR), when the detection rate of trout was comparable between cameras (UVS) and from streambanks (VSS). During the high-flow regime (HFR), streambank observations were, however, much less effective. Thus, the two visual methods seem to be reliable and comparable during the drought period and provide a similar description of the pattern of occurrence.

This is good news, given that visual surveys from streambanks are generally the simplest, least invasive and most cost-effective method for observing fish, as they require no capture of individuals, no diving, no or minimal passage into the water and very little equipment. Hiking with light equipment to explore stream stretches as far as possible seems to be a very practical method when sampling headwater streams is challenging due to limited access areas and rugged terrain [27]. Factors such as the time taken to survey a site and the weight of sampling equipment are very important when considering which methods should be used. Underwater video equipment has become compact and easily transportable to remote sampling sites [78]. However, it is important to consider that post-processing in the laboratory can be prohibitively time-consuming [16,35,79].

GLM analysis showed the effect of some habitat variables on the occurrence of the Mediterranean trout, as discussed below. The best multivariate model applied to the UVS data identified the volume of pools and the percentage cover of submerged macrophytes as the most important environmental variables that affect fish detection probability. Specifically, the likelihood of detecting trout with underwater cameras was positively correlated with pool size. This could be explained by the fact that trout exhibit a high preference for habitat pools [67], and bigger pools are important thermal refuges where fish tend to aggregate, particularly during drought periods [80]. This effect could thus be attributed to a higher number of trout being present in larger pools. However, it is important to note that the rarity of trout could increase the chances of missing their presence, and imperfect detection can become inflated in the case of a discontinuous or patchy distribution [81]. The second effect identified, although much weaker, was the reduction in detection probability with a higher percentage cover of submerged macrophytes in the pool. Our results are consistent with previous studies, and our hypothesis is that the presence of submerged macrophytes has a negative effect on fish detection [34]. This result may be due to submerged vegetation obscuring the camera’s field of view, making it difficult to identify fish clearly. Our findings also apparently contradict those of most published studies on the use of underwater cameras in freshwater habitats that have suggested turbidity as a limiting factor [20,22,35]. However, it should be noted that the conditions for detecting trout during this study were optimal, and the water was generally clear (0.1–1.4 NTU), making species identification relatively easy. Furthermore, despite the fact that turbulence rates were often higher during the HFR, they did not appear to have affected the detection probability of trout with underwater cameras.

On the other hand, the analysis of data coming from VSS shows a well-supported negative effect of high turbulence rates on pool occupancy. It is likely that the higher the turbulence rate, the more glaring appears on the surface water, making it difficult to observe the stream underwater. This effect could thus be ascribed to the lower detectability of trout with a higher turbulence rate. In cases in which glare from the water’s surface reduces visibility, polarized sunglasses are strongly advised to increase visibility from streambanks [15,33]. Bankside observations are particularly effective towards the end of the dry season and can be employed with minimal flow to monitor occupancy rates of persistent pools and assess stream fragmentation.

It is important to acknowledge that other complex and multifaceted factors could potentially influence the detection probability of trout, and our study may not have fully captured the extent of their influence. Indeed, the detection probability of a species can vary significantly depending on factors such as the sampling methods and effort employed, fish size, physical habitat characteristics, local density and seasonal or behavioral patterns [82]. Additionally, the probability of detecting a species is a combination of the sampling method performance and environmental characteristics, which also play a role in determining its habitat requirements.

In this context, certain environmental parameters such as fast-flowing conditions, water temperature, dissolved oxygen levels, substrate and organic covers are well-known factors that favor the presence and abundance of trout [83,84].

## 5. Conclusions

In conclusion, our study confirms that visual surveys can be a useful method for detecting Mediterranean trout in small streams. Additionally, this is the first study to compare underwater visual surveys (UVS) with visual observation from streambanks (VSS) for assessing trout occurrence. The comparison revealed that underwater camera surveys perform comparably to visual surveys from streambanks during low-flow regimes. Both methods, UVS and VSS, present notable advantages in terms of their simplicity, versatility, cost-effectiveness and non-invasiveness. However, it is important to recognize the limitations associated with visual survey techniques. Fish observation from streambanks may create difficulties in the taxonomic identification and counting of small fish. Therefore, conducting bankside visual observation may necessitate specialized expertise in fish behaviors, skills that could be possessed by recreational anglers. Similarly, beyond the cost of camera equipment, underwater video surveys appear to be a cost-effective way to capture reliable information about trout occurrence and could also be used to gather information about ecological interactions and fish behavior, without the observer’s potentially disruptive presence [28,34,85]. The lightweight design and easy use of underwater cameras make this method particularly well-suited for citizen science projects with the collaboration of civil society, including students, clubs and others, in data collection and the monitoring of trends in rare or threatened species. On the other hand, expertise is required for the video analysis to reliably count and identify species. In general, visual survey estimates can be an important tool for fish biologists and conservationists to accurately estimate trout occupancy in small streams and could be considered more extensively for monitoring programs of endangered fish species in small intermittent streams. Moreover, visual survey observation could also be employed in detecting invasive fish species by supporting environmental DNA monitoring (eDNA).

However, we acknowledge the need for further studies to evaluate and compare the relative performance of electrofishing and visual surveys in estimating trout abundance in small streams as well as the need to explore the role of additional environmental variables on the distribution and abundance of Mediterranean trout.

## Figures and Tables

**Table 1:** Results of model selection based on AICc of univariate GLM analysis of Mediterranean trout occurrence data obtained with underwater visual survey (UVS) and visual survey from streambanks (VSS). Likelihood ratio test (LRT) used to assess the differences among candidate models and null model (M0). Best models in bold. Asterisks indicate significant differences (* *p* < 0.05, ** *p* < 0.01, *** *p* < 0.001).

**Underwater Visual Survey (UVS)**									
**Rank**	**Model effect**	**Ka**	**AICc**	**ΔAICc**	**Dev. Expl. (%)**	**Slope**	**SE**	***p***	**LRT**
**1**	**Volume (m^3^)**	**2**	**109.598**	**0**	**10.00%**	**0.03**	**0.01**	0.0042 **	0.0005 ***
**2**	**Submerged_macrophyte (%)**	**2**	**116.132**	**6.534081**	**4.72%**	**−0.15**	**0.07**	0.0491 *	0.01844 *
**3**	**Boulder (%)**	**2**	**116.274**	**6.675573**	**4.60%**	**−0.02**	**0.009**	0.0233 *	0.01999 *
4	Riparian_vegetation (%)	2	118.789	9.190578	2.46%	0.05	0.03	0.101	
5	M0	1	119.589	9.990974					
6	Green_Algae (%)	2	121.541	11.94337					
7	Roots (%)	2	121.604	12.00592					
8	Turbulence_rate (High, Medium, Low)	2	121.67	12.07229					
9	Turbidity (NTU)	2	121.685	12.08668					
**Visual Survey from Streambanks (VSS)**									
**Rank**	**Model effect**	**Ka**	**AICc**	**ΔAICc**	**Dev. Expl. (%)**	**Slope**	**SE**	***p***	**LRT**
**1**	**Turbulence_rate (High, Medium, Low)**	**2**	**80.1**	**0**	**29.03%**	**−2.42**	**0.62**	0.0001 ***	2.4 × 10^8^ ***
**2**	**Turbidity (NTU)**	**2**	**100.34**	**20.24**	**10.11%**	**2.19**	**0.7**	0.0018 **	0.0009977 ***
**3**	**Boulder (%)**	**2**	**107.16**	**27.07**	**3.73%**	**−0.02**	**0.01**	**0.0555**	0.0455 *
4	Riparian_vegetation (%)	2	108.21	28.11	2.75%	0.06	0.03	0.087	
5	Submerged_macrophyte (%)	2	108.58	28.48	2.41%	−0.12	0.09	0.192	
6	M0	1	109.07	28.29					
7	Roots (%)	2							
8	Volume (m^3^)	2							
9	Green_Algae (%)	2							

**Table 2:** Results of model selection based on AICc of multivariate GLMs analysis of Mediterranean trout occurrence data obtained with underwater visual survey (UVS) and visual survey from streambanks (VSS). Best models in bold.

**Underwater Visual Survey (UVS)**				
**Rank**	**Model Decription**	**AICc**	**ΔAICc**	**Weights (w_i_)**
1	**UVS ~ 1 + Volume + Submerged_Macrophytes**	**106.70**	**0.00**	**0.47**
2	UVS ~ 1 + Volume + Boulder + Submerged_Macrophytes	107.54	0.83	0.31
3	UVS ~ 1 + Volume	109.60	2.90	0.11
4	UVS ~ 1 + Volume + Boulder	110.76	4.06	0.06
5	UVS ~ 1 + Boulder + Submerged_Macrophytes	112.14	5.44	0.03
6	UVS ~ 1 + Submerged_Macrophytes	116.13	9.43	0.00
7	UVS ~ 1 + Boulder	116.27	9.57	0.00
8	UVS ~ 1	119.59	12.89	0.00
**Visual Survey from Streambanks (VSS)**				
**Rank**	**Model Decription**	**AICc**	**ΔAICc**	**Weights (w_i_)**
1	VSS ~ 1 + Turbulence_rate + Boulder	79.16	0.00	0.43
**2**	**VSS ~ 1 + Turbulence_rate**	**80.10**	**0.93**	**0.27**
3	VSS ~ 1 + Turbulence_rate + Turbidity + Boulder	80.64	1.48	0.20
4	VSS ~ 1 + Turbulence_rate + Turbidity	82.03	2.87	0.10
5	VSS ~ 1 + Turbidity + Boulder	99.75	20.59	0. 01
6	VSS ~ 1 + Turbidity	100.34	21.17	0.01
7	VSS ~ 1 + Boulder	107.17	28.01	0.00
8	VSS ~ 1	1109.07	1029.91	0.00

**Table 3:** Model-averaged importance of main factors for underwater visual survey (UVS) and visual survey from streambanks (VSS). Cut-off is set at 0.8 to explore the most important predictors (in bold).

Underwater Visual Survey (UVS)			
Effect	Estimate	Unconditional Variance	Importance
Boulder	−0.0053	7.37 × 10^5^	0.4097
**Submerged_Macrophytes**	**−0.1279**	**8.82 × 10^3^**	**0.8216**
**Volume**	**0.0278**	**1.49 × 10^4^**	**0.9598**
Visual Survey from Streambanks (VSS)			
Turbidity	−0.2200	0.2480	0.3054
Boulder	−0.0136	0.0002	0.6305
**Turbulence rate**	**−2.5892**	**0.5194**	**0.9999**

## Data Availability

The data used in this manuscript will be made available from the corresponding authors upon reasonable request.

## Supplementary Materials

The following supporting information can be downloaded at: https://www.mdpi.com/article/10.3390/biology12071000/s1, Table S1: Descriptive statistics of pool characteristics surveyed in Piras stream during the two different flow regimes. K-W test (* *p* < 0.05, ** *p* < 0.01, *** *p* < 0.001); Table S2: List of variables measured for each pool; Table S3: Results of collinearity test.
