# Supplementary material for: Exploring Less Invasive Visual Surveys to Assess the Spatial Distribution of Endangered Mediterranean Trout Population in a Small Intermittent Stream
Source: Biology (Basel). 2023 Jul 13;12(7):1000. doi: 10.3390/biology12071000 (PMC10376087; doi:10.3390/biology12071000)
Supplement: Supplementary file 1 [file biology-12-01000-s001.zip › biology-2485964-supplementary.pdf]

Supplementary Materials

Table S1. Descriptive statistics of pools characteristics surveyed in Piras stream during the two different flow regimes. K-W test (*p< 0.05, **p< 0.01, ***p< 0.001).

Variables		High flow regime (HFR)			Low flow regime (LFR)		
		**Mean±SE**	**Median**	**Range**	**Mean±SE**	**Median**	**Range**
Pool size	Pool_Length (m) (*)	7.92±0.48	8	3-17	6.77±0.49	7	03-17
	Pool_Width (m) (**)	4.34±0.23	4	1.5-9	3.49±0.26	3.3	0.1-ago
	Depth (m)	0.84±0.06	0.7	0.3-1.8	0.67±0.06	0.6	0.25-1.74
	Volume (m^3) (*)	35.89±5.32	16.9	2.8-140	21.73±3.88	9	1.75-93.26
Chemical-Physical characteristics of water	T_H_2O (°C) (***)	9.83±0.10	9.5	8.9-10.8	16.35±0.09	16.3	15-18
	O2_mgl (mg/l) (***)	10.54±0.03	10.6	10.2-10.9	8.27±0.17	8.6	4-9.2
	Turbidity (NTU) (***)	0.33±0.02	0.3	0.1-0.9	0.86±0.04	0.8	0.4-1.4
Substrate	Bedrock (%)	15.53±2.9	10	0-80	16.28±3.25	10	0-90
	Boulder (%)	32.02±3.57	30	0-80	27.43±3.87	20	0-80
	Cobble (%)	32.23±3.01	30	0-85	35.12±3.45	30	0-85
	Gravel (%)	17.44±2.32	10	0-55	17.82±2.59	10	0-55
	Sand (%)	2.76±0.89	0	0-30	3.33±1.06	0	0-30
	Silt (%)	0	0	0	0	0	0
Organic cover	Emergent_Macrophytes (%)	0.95±0.39	0	0-10	0.51±0.30	0	0-10
	Submerged_Macrophytes (%)	1.06±2.32	0	0-10	1.92±0.81	0	0-20
	Periphyton (%) (***)	69.36±3.65	75	10-100	50.89±4.40	60	5-100
	Green_Algae (%)	7.34±2.63	0	0-80	12.05±3.44	0	0-75
	Roots (%)	9.89±1.23	10	0-30	8.59±1.23	10	0-30
	Riparian_vegetation (%)	5.35±0.96	5	0-35	5.52±1.05	0	0-20
	XYLAL (%) (***)	4.57±1.29	0	0-50	11.41±1.57	10	0-40
	FPOM (%) (***)	1.38±0.66	0	0-25	9.61±2.02	5	0-60

Table S2. List of variables measured for each pool.

	Variables	Category	Descrition and units
Sampling Seasons	Flow_regime	Factor	Highest and lowest flows regime (HIGH and LOW)
Pool size	Pool_Length	Continuous	Pool Length (m)
	Pool_Width	Continuous	Average pool width (m)
	Depth	Continuous	Maximum value of pool depth (m)
	Volume	Continuous	Pool volume [length (m) × average width (m) × average depth (m)] (m^3)
Chimical-Physical characteristics of water	T_H2O	Continuous	Average water temperature recorded during the samplings (°C)
	O2_mgl	Continuous	Oxygen concentration recorded during the samplings (O2, mg l^{-1})
	Turbulence_rate	Factor	Water turbulence: Low (LTR), Medium (MTR) and High(HTR)
	Turbidity	Continuous	Water nephelometric turbidity recorded during the samplings (NTU)
Substrate	Bedrock	Continuous	Exposed bedrock along the streambed (%)
	Boulder	Continuous	Boulder (more than 304.0 mm) (%)
	Cobble	Continuous	Cobble (76.1–304.0 mm) (%)
	Gravel	Continuous	Gravel (4.8–76.0 mm) (%)
	Sand	Continuous	Sand (0.8–4.7 mm) (%)
	Silt	Continuous	Silt (particle size less than 0.8 mm) (%)
Organic cover	Emergent_Macrophytes	Continuous	Erbaceus macrophytes (%)
	Submerged_Macrophytes	Continuous	Subaqueous macrophytes partly above the water or completely underwater (%)
	Periphyton	Continuous	Microalgae that cover the substrate of streambad (%)
	Green_Algae	Continuous	Filamentous green algae (%)
	Roots	Continuous	Roots of the riparian vegetation (%)
	Riparian_vegetation	Continuous	Vegetation at riverside extending at least 20 cm into the channel (%)
	XYLAL	Continuous	Dead wood, non-living tree tranks and branches (%)
	FPOM	Continuous	Fine particular organic material (%)

Table S3. Result of collinearity test.

**Selected variables**	**Variance inflation factor (VIF)**
Volume	1.068305
pH	1.462694
Turbidity	1.629437
Green_Algae	1.157672
Submerged_Macrophytes	1.303739
Roots	1.207014
Riparian_vegetation	1.240164
